# Silymarin Attenuates Nicotine-Associated Testicular Degeneration and Systemic Oxidative and Inflammatory Changes in Mice

**DOI:** 10.3390/molecules31203779

**Published:** 2026-10-21

**Authors:** Abdulsamed Kükürt, Mahmut Karapehlivan, Hasan Özen, İnan Kaya, Tolga Kasacı, Haci Ahmet Deveci, Elnare Cevad Erkan, Metin Öğün, Ayla Özcan, Ömer Faruk Başer, Recai Aci, Hilmi Nuhoğlu

**Affiliations:** 1 Department of Biochemistry, Faculty of Veterinary Medicine, Kafkas University, 36100 Kars, Türkiye; 2 Department of Medical Biochemistry, Faculty of Medicine, Kafkas University, 36100 Kars, Türkiye; mkarapehlivan@gmail.com (M.K.); dr70194@gmail.com (T.K.); metinogun@hotmail.com (M.Ö.); omerfarukbaser@kafkas.edu.tr (Ö.F.B.); 3 Department of Pathology, Department of Preclinical Sciences, Faculty of Veterinary Medicine, Balıkesir University, 10100 Balıkesir, Türkiye; hasanozen@hotmail.com; 4 Department of Biology, Division of Biochemistry and Molecular Biology, Faculty of Arts and Sciences, Kafkas University, 36100 Kars, Türkiye; inankaya_@hotmail.com; 5 Department of Nutrition and Dietetics, Faculty of Health Sciences, Gaziantep University, 27100 Gaziantep, Türkiye; h.ahmet_deveci@hotmail.com; 6 Emergency Department, Terme State Hospital, 55600 Samsun, Türkiye; ekaelish@hotmail.com; 7 Medical Laboratory Techniques Program, Department of Medical Services and Techniques, Vocational School of Health Services, Gazi University, 06170 Ankara, Türkiye; aylabicer@hotmail.com; 8 Söke Health Services Vocational School, Aydın Adnan Menderes University, 09100 Aydın, Türkiye; recaiaci@adu.edu.tr; 9 Department of Pathology, Faculty of Veterinary Medicine, Kafkas University, 36100 Kars, Türkiye; hilmi.nuhoglu@kafkas.edu.tr

**Keywords:** nicotine, silymarin, male reproductive toxicity, testicular degeneration, oxidative stress, NF-κB, apoptosis

## Abstract

Nicotine exposure can disrupt redox homeostasis and damage the male reproductive system. This study evaluated short-term nicotine-associated systemic and testicular changes and examined whether concurrent silymarin treatment attenuated these alterations. Twenty-eight adult male Swiss albino mice were randomized to control, nicotine, silymarin, and nicotine plus silymarin groups (*n* = 7 each). Nicotine (1.5 mg/kg/day) and silymarin (100 mg/kg/day) were administered intraperitoneally for seven days. Whole-blood glutathione, plasma malondialdehyde, superoxide dismutase, nitric oxide, paraoxonase-1, total sialic acid, lipid profile, caspase-3, and nuclear factor kappa B, testicular redox markers, and testicular histomorphology were assessed. Nicotine was associated with lower blood glutathione and plasma superoxide dismutase and paraoxonase-1 activities, higher plasma malondialdehyde, nitric oxide, total sialic acid, caspase-3, and nuclear factor kappa B values, and an unfavorable testicular redox profile (all *p* < 0.001). Degenerative changes were also observed in seminiferous tubules and Leydig cells. Concurrent silymarin treatment attenuated most biochemical alterations and was associated with less severe histological injury, although several markers did not fully return to control values. These findings support a partial protective association under the conditions tested; reproductive function was not directly assessed.

## 1. Introduction

The male reproductive system is particularly susceptible to disrupted redox balance because of rapid cell division, reactive species generated during steroidogenesis, and the high polyunsaturated fatty acid content of germ-cell membranes. Physiological concentrations of reactive oxygen species contribute to sperm maturation and capacitation, whereas production that exceeds antioxidant capacity may cause lipid peroxidation, protein oxidation, and DNA damage [1].

Nicotine is the principal addictive alkaloid in tobacco products and may produce oxidative injury in male reproductive tissue independently of other constituents of tobacco smoke. Experimental studies have associated nicotine exposure with reduced testicular antioxidant enzyme activities, increased malondialdehyde (MDA) and nitric oxide (NO), impaired sperm count and motility, and degenerative changes in seminiferous-tubule architecture [2,3,4,5]. These observations identify oxidative and nitrosative stress as important components of nicotine-associated testicular toxicity.

Silymarin, extracted from Silybum marianum seeds, is a mixture of flavonolignans that includes silibinin, silychristin, and silydianin. It has been reported to limit free-radical formation, support cellular glutathione pools and antioxidant enzyme responses, preserve mitochondrial integrity, and influence nuclear factor erythroid 2-related factor 2 signaling [6]. Experimental reproductive studies have also suggested that silymarin can improve testicular redox balance, sperm parameters, and histological structure in several toxic and metabolic injury models [7,8,9].

Although previous investigations, such as those by Rahimi-Madiseh et al. [10], have explored the protective efficiency of silymarin against nicotine-induced reproductive toxicity by focusing on long-term sperm parameters and in vitro fertilization outcomes, the acute windows of systemic versus localized testicular tissue-level oxidative adaptations remain inadequately characterized. Short-term exposure dynamics are critical to understanding the immediate, early-phase soft-tissue damage and the prompt therapeutic windows of antioxidant interventions before permanent structural degradation occurs. Evidence integrating circulating redox indicators, peripheral inflammation- and apoptosis-related markers, and targeted testicular tissue defenses within an acute timeframe remains limited. Therefore, the present study evaluated the immediate consequences of a short-term, seven-day nicotine administration protocol on both circulating and testicular redox homeostasis in mice, while systematically defining the capacity of concurrent silymarin treatment to mitigate these acute localized alterations.

## 2. Results

### 2.1. Blood and Plasma Biochemical Findings

**Figure 1:**
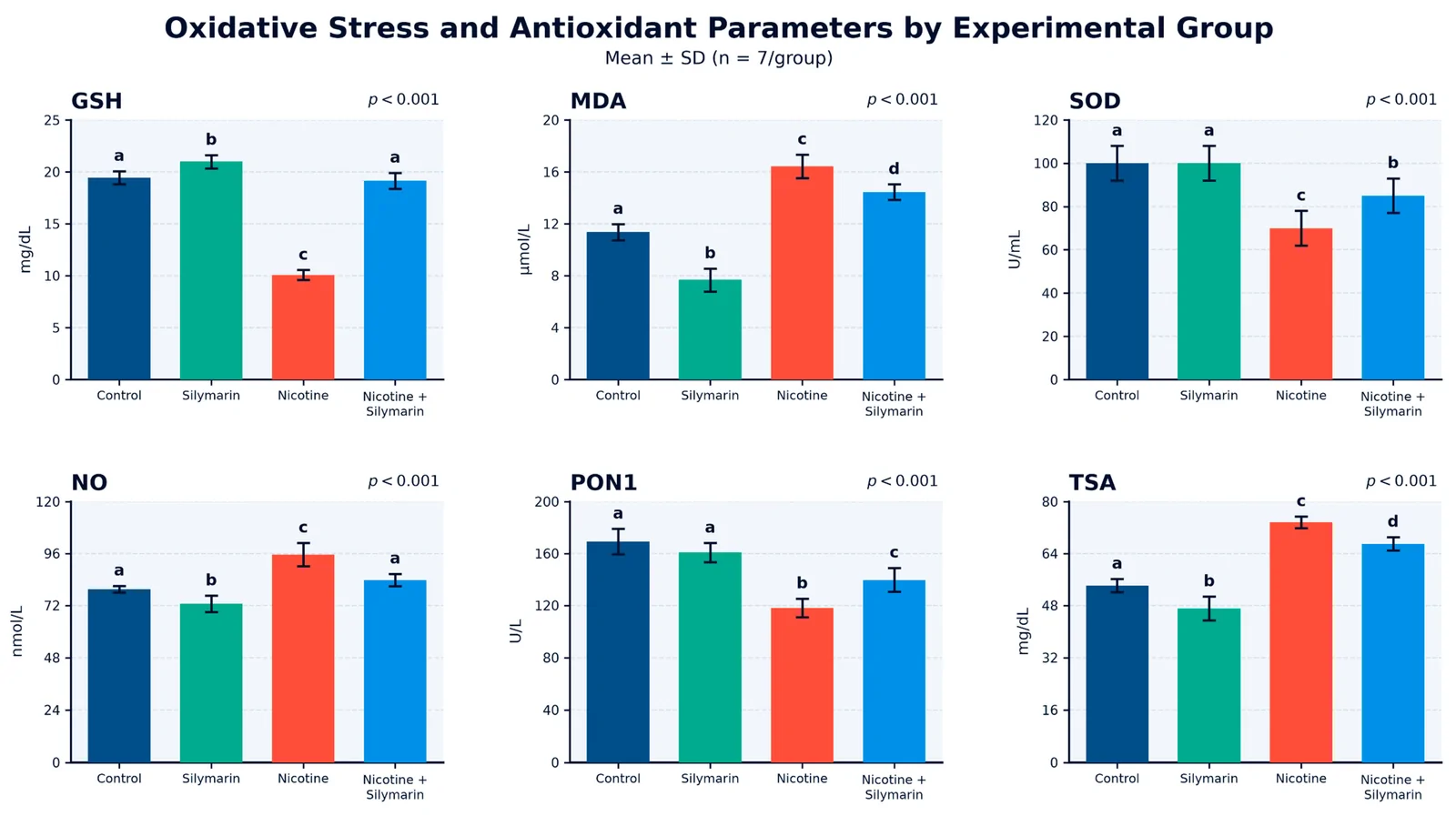
Blood and plasma oxidative stress and antioxidant parameters across the experimental groups. Panels show whole-blood glutathione (GSH) content, plasma malondialdehyde (MDA) concentrations, plasma superoxide dismutase (SOD) enzyme activities, plasma total nitrate and nitrite (NO) levels, plasma paraoxonase-1 (PON1) enzyme activities, and plasma total sialic acid (TSA) concentrations in the control, silymarin, nicotine, and nicotine plus silymarin groups. Data are presented as mean ± standard deviation (*n* = 7) per group. Within each panel, bars marked with different superscript letters indicate statistically significant differences (*p* < 0.05).

Whole-blood GSH: Reduced glutathione levels differed markedly among the four experimental groups (*p* = 5.32 × 10^−21^). Post hoc pairwise comparisons revealed significant differences between the nicotine group and all other groups, reflecting a profound depletion of circulating glutathione following nicotine exposure (*p* < 0.001). The control and nicotine plus silymarin groups did not differ significantly (*p* = 0.810), indicating a remarkable statistical recovery of circulating GSH with the combined treatment (Figure 1).

Plasma MDA: The overall group effect on malondialdehyde concentrations was highly significant (*p* = 1.94 × 10^−16^), with significant variations confirmed across all reciprocal pairwise comparisons (*p* < 0.001). These findings indicate that nicotine exposure markedly accelerated lipid peroxidation, whereas concurrent silymarin administration significantly reduced MDA levels relative to nicotine alone, although it did not restore them fully to the baseline control values (Figure 1).

Plasma SOD: Nicotine exposure significantly depressed systemic superoxide dismutase enzyme activity relative to the control group (*p* = 2.45 × 10^−6^). Concurrent silymarin treatment significantly counteracted this suppression compared with nicotine alone (*p* < 0.05), although the enzymatic activity remained lower than the healthy control values (*p* < 0.05; Figure 1).

Plasma NO: Total nitrate and nitrite levels differed significantly among the experimental groups (*p* = 3.11 × 10^−10^). Post hoc pairwise comparisons revealed significant variations between all treatment groups, except for the comparison between the control and nicotine plus silymarin groups (*p* = 0.171). Accordingly, the concurrent treatment successfully mitigated the nicotine-associated surge in NO, reducing it to a level that was statistically comparable to the healthy controls (Figure 1).

Plasma PON1: Paraoxonase-1 activity demonstrated significant differences among the evaluated groups (*p* = 1.40 × 10^−10^). While no significant variance was observed between the control and silymarin-alone groups (*p* = 0.268), nicotine exposure induced a severe drop in PON1 activity. Concurrent silymarin treatment significantly boosted enzyme activity relative to the nicotine group, although it still remained below the baseline control threshold (*p* < 0.05; Figure 1).

Plasma TSA: Total sialic acid levels differed significantly among the experimental groups (*p* = 4.62 × 10^−16^), with highly significant differences confirmed across all reciprocal pairwise comparisons (*p* < 0.001). Nicotine exposure markedly elevated plasma TSA concentrations, whereas concurrent silymarin administration produced a significant but partial reduction relative to the nicotine-alone group (Figure 1).

**Figure 2:**
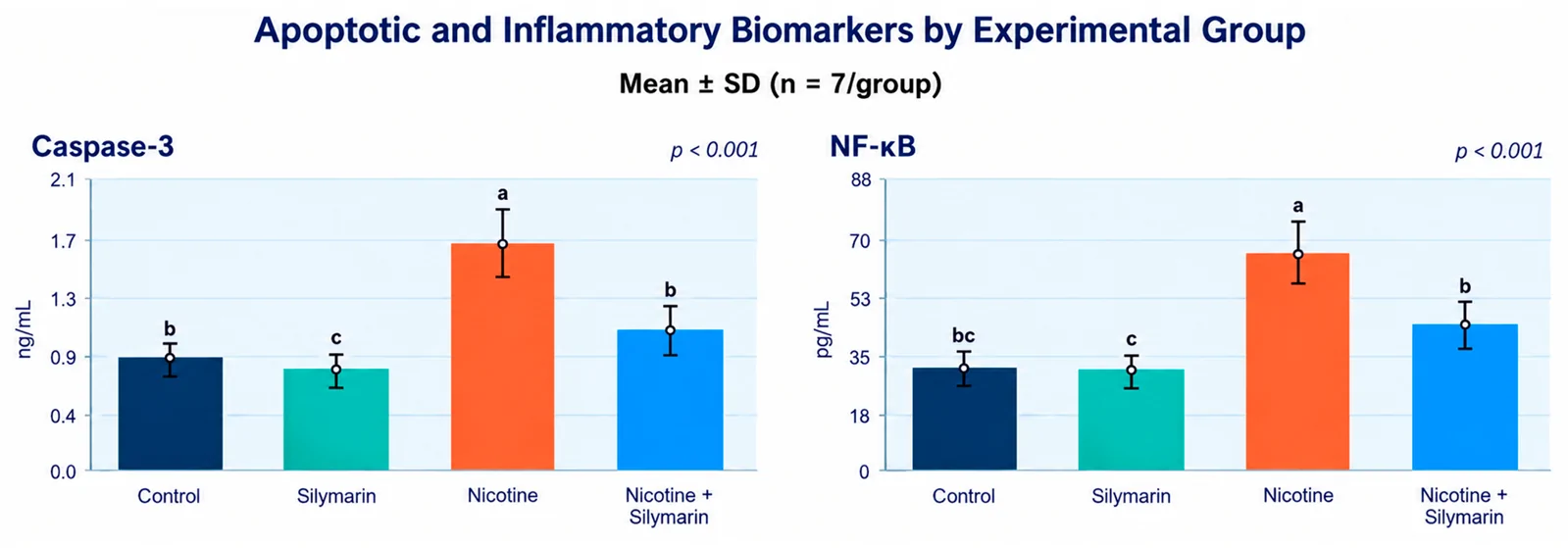
Peripheral apoptotic and inflammatory biomarkers across experimental groups. Panels display plasma caspase-3 and nuclear factor kappa B (NF-κB) concentrations determined by ELISA in the control, silymarin, nicotine, and nicotine plus silymarin groups. Data are presented as mean ± standard deviation (*n* = 7) per group. Bars sharing different superscript letters indicate statistically significant differences (*p* < 0.05).

Plasma caspase-3: Systemic caspase-3 concentrations differed significantly among the evaluated groups (F(3,24) = 46.65, *p* = 3.63 × 10^−10^). Nicotine exposure induced a prominent elevation in caspase-3 values compared to both the control (*p* = 3.95 × 10^−9^) and silymarin groups (*p* = 9.52 × 10^−10^). Concurrent silymarin treatment significantly attenuated this apoptotic marker compared to the nicotine group (*p* = 7.22 × 10^−7^), though values remained higher than the silymarin-alone group (*p* = 0.0193). No significant variance was noted between the control group and either the silymarin (*p* = 0.8806) or combined treatment groups (*p* = 0.0944; Figure 2).

Plasma NF-κB: Nuclear factor kappa B concentrations demonstrated significant inter-group variations (F(3,24) = 31.84, *p* = 1.56 × 10^−8^). Values in the nicotine group were significantly higher than those in the control (*p* = 7.80 × 10^−8^) and silymarin groups (*p* = 5.68 × 10^−8^). Combined treatment effectively lowered NF-κB levels relative to nicotine alone (*p* = 4.00 × 10^−5^), although the levels remained higher than in the silymarin-alone group (*p* = 0.0424). No significant differences were observed for the control group versus the silymarin (*p* = 0.9988) or nicotine plus silymarin groups (*p* = 0.0581; Figure 2).

**Figure 3:**
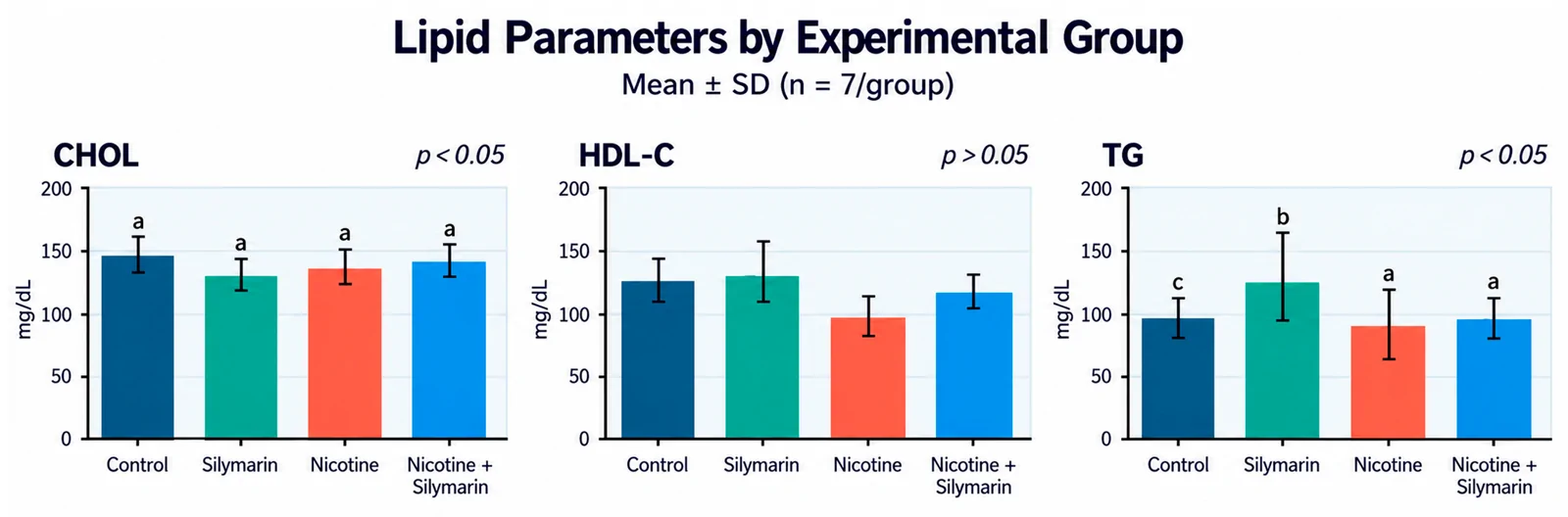
Plasma lipid profile parameters (total cholesterol [CHOL], high-density lipoprotein cholesterol [HDL-C], and triglyceride [TG] concentrations) in the evaluated groups. Data are shown as mean ± standard deviation (*n* = 7 per group). Different superscript letters indicate statistically significant differences (*p* < 0.05); groups without letters or sharing identical markers display no significant variance (*p* > 0.05).

Plasma CHOL: Total cholesterol levels did not differ significantly among the experimental groups (*p* = 0.573), and no significant differences were detected in reciprocal pairwise comparisons (Figure 3).

Plasma TG: Triglyceride levels showed a significant overall group effect (*p* = 0.0136). The silymarin-alone group exhibited significantly higher values compared to the control (*p* = 0.0446), nicotine (*p* = 0.0248), and nicotine plus silymarin groups (*p* = 0.0320); no other significant variations were found among the pairs (Figure 3).

Plasma HDL-C: High-density lipoprotein cholesterol concentrations did not differ significantly among the groups (*p* = 0.0682), and none of the subsequent pairwise comparisons reached statistical significance (Figure 3).

### 2.2. Testicular Oxidative-Stress and Antioxidant-Defense Findings

**Figure 4:**
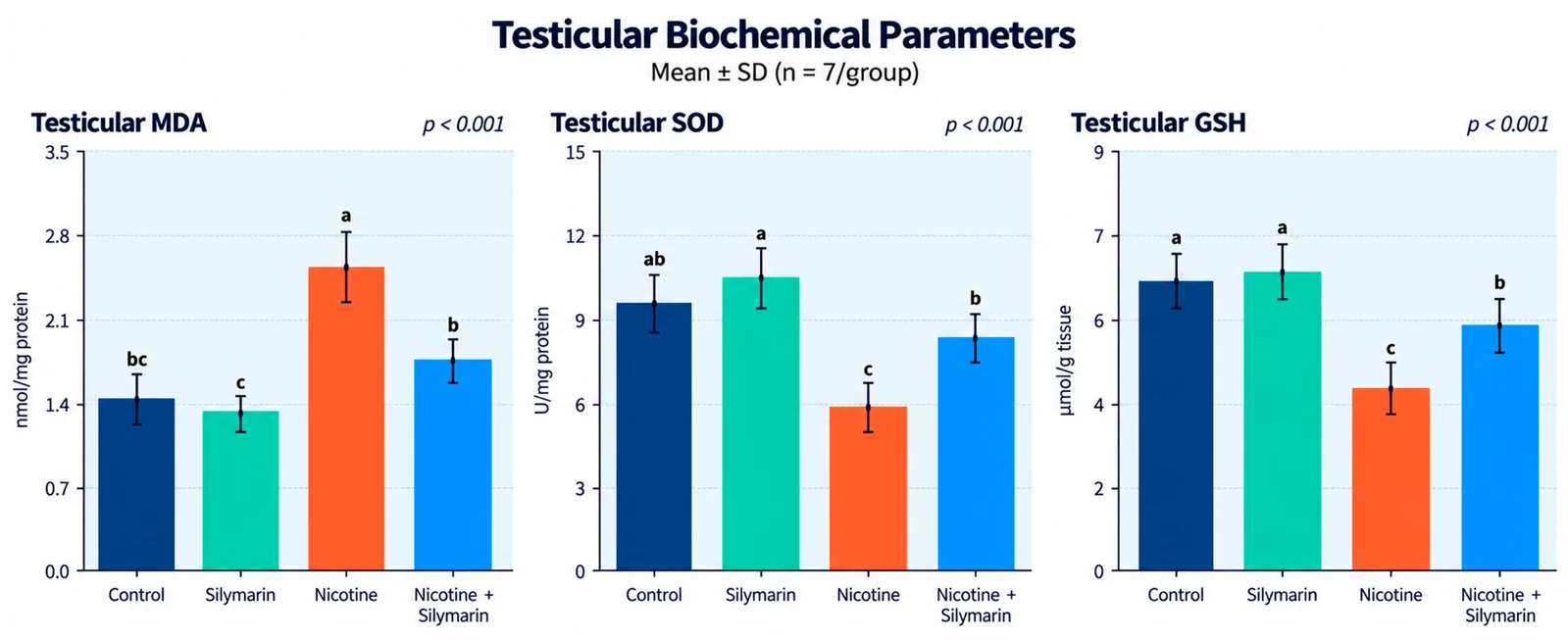
Testicular tissue malondialdehyde (MDA) concentrations, superoxide dismutase (SOD) enzyme activities, and reduced glutathione (GSH) content following spectrophotometric determination and total tissue protein normalization in the experimental groups. Data represent mean ± standard deviation (*n* = 7 per group). Within each parameter, bars marked with different superscript letters are statistically significant (*p* < 0.05).

Testicular MDA: Malondialdehyde differed significantly among groups (F(3,24) = 38.48, *p* = 2.50 × 10^−9^). The nicotine group had higher MDA than the control (*p* = 3.03 × 10^−8^), silymarin (*p* < 0.001), and nicotine plus silymarin groups (*p* < 0.001). The nicotine plus silymarin group remained higher than the silymarin group (*p* = 0.0237), whereas the control group did not differ from silymarin (*p* = 0.8243) or nicotine plus silymarin (*p* = 0.1424; Figure 4).

Testicular SOD: Superoxide dismutase activity differed significantly among groups (F(3,24) = 20.98, *p* = 6.82 × 10^−7^). Activity in the nicotine group was lower than in the control (*p* = 1.67 × 10^−5^) and silymarin groups (*p* = 6.52 × 10^−7^). The nicotine plus silymarin group had higher activity than the nicotine group (*p* = 0.0028) but lower activity than the silymarin group (*p* = 0.0108). Control did not differ from silymarin (*p* = 0.5279) or nicotine plus silymarin (*p* = 0.1935; Figure 4).

Testicular GSH: Reduced glutathione differed significantly among groups (F(3,24) = 23.01, *p* < 0.001). The nicotine group had lower GSH than the control (*p* = 2.28 × 10^−6^), silymarin (*p* < 0.001), and nicotine plus silymarin groups (*p* = 0.0058). Although combined treatment increased GSH relative to nicotine alone, values remained lower than in the control (*p* = 0.0191) and silymarin groups (*p* = 0.0071). Control and silymarin did not differ (*p* = 0.9742; Figure 4).

### 2.3. Histopathological Findings

**Figure 5:**
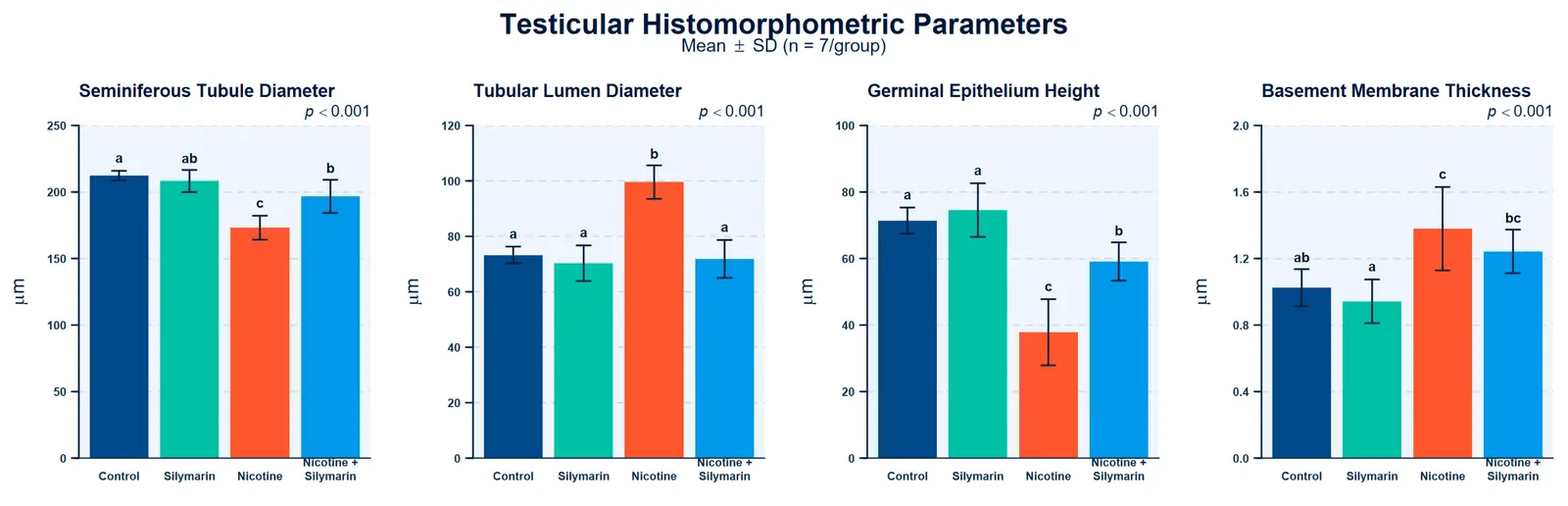
Testicular histomorphometric parameters, including seminiferous tubule diameter, tubular lumen diameter, germinal epithelium height, and basement membrane thickness, across the experimental groups. Data are presented as mean ± standard deviation (*n* = 7 per group). Within each parameter, bars marked with different superscript letters differ significantly according to Tukey’s HSD test (*p* < 0.05).

Seminiferous tubule diameter: The overall group effect was significant (F(3,24) = 27.10, *p* = 7.04 × 10^−8^). The nicotine group had a smaller mean diameter than the control (*p* = 1.19 × 10^−7^), silymarin (*p* = 7.96 × 10^−7^), and nicotine plus silymarin groups (*p* = 0.000273). The nicotine plus silymarin group remained lower than the control group (*p* = 0.0162) but did not differ from the silymarin group (*p* = 0.101). Control and silymarin did not differ (*p* = 0.831; Figure 5).

Tubular lumen diameter: Lumen diameter differed significantly among groups (F(3,24) = 40.67, *p* = 1.44 × 10^−9^). The nicotine group had a larger lumen diameter than the control (*p* = 5.95 × 10^−8^), silymarin (*p* = 8.18 × 10^−9^), and nicotine plus silymarin groups (*p* = 2.28 × 10^−8^). Control, silymarin, and nicotine plus silymarin did not differ from one another (all *p* > 0.05; Figure 5).

Germinal epithelium height: The overall group effect was significant (F(3,24) = 36.52, *p* = 4.17 × 10^−9^). The nicotine group had a lower germinal epithelium height than the control (*p* = 4.81 × 10^−8^), silymarin (*p* = 9.02 × 10^−9^), and nicotine plus silymarin groups (*p* = 7.30 × 10^−5^). The nicotine plus silymarin group remained lower than both the control (*p* = 0.0208) and silymarin groups (*p* = 0.00303). Control and silymarin did not differ (*p* = 0.851; Figure 5).

Basement membrane thickness: Basement membrane thickness differed significantly among groups (F(3,24) = 10.19, *p* = 0.000163). The nicotine group had a thicker basement membrane than the control (*p* = 0.00273) and silymarin groups (*p* = 0.000275) but did not differ from the nicotine plus silymarin group (*p* = 0.431). The nicotine plus silymarin group was higher than the silymarin group (*p* = 0.0121) but did not differ significantly from the control group (*p* = 0.0911). Control and silymarin did not differ (*p* = 0.794; Figure 5).

**Figure 6:**
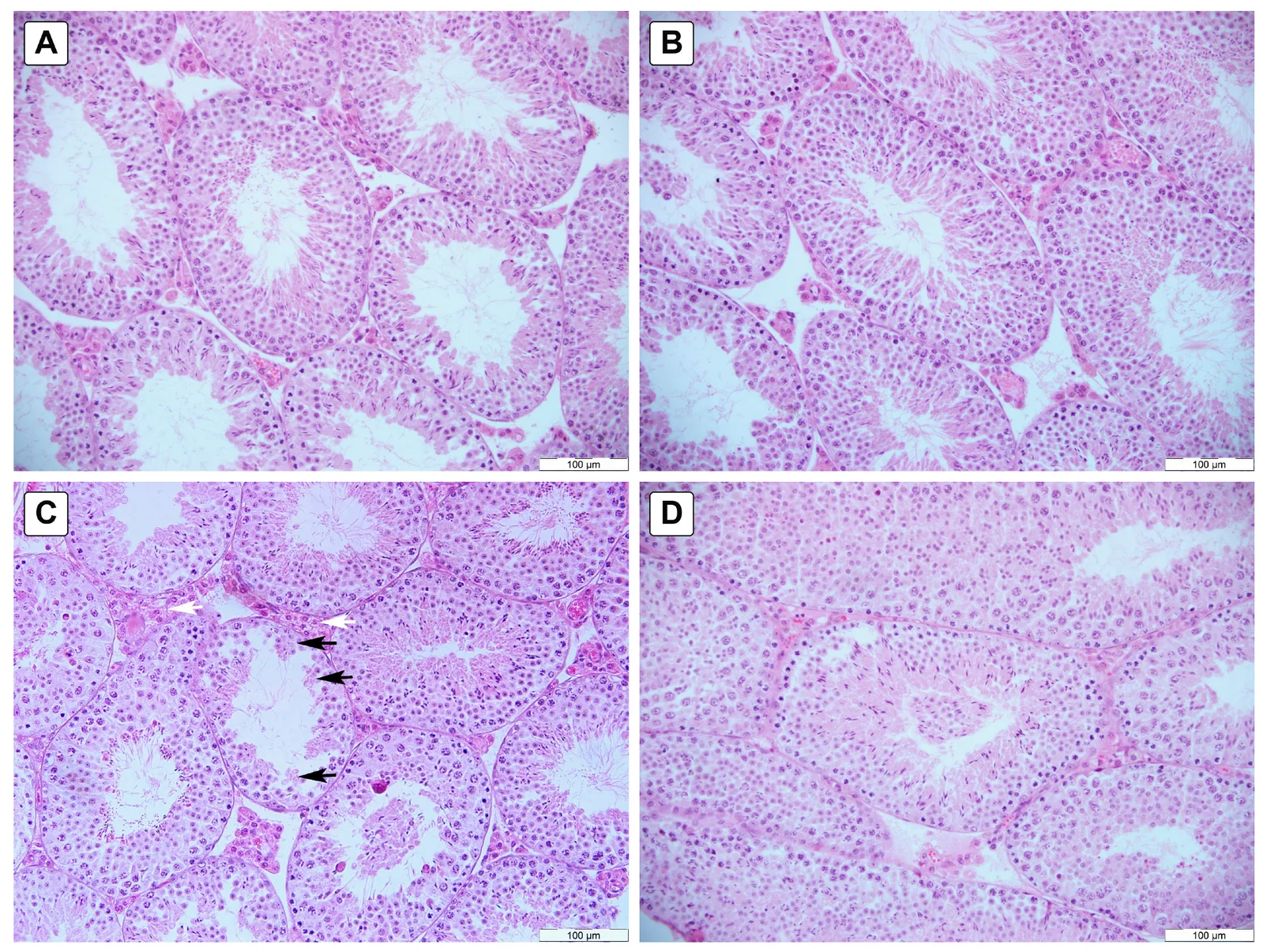
Histopathological appearance of testicular tissue. (**A**) Control group: normal testicular histomorphology. (**B**) Silymarin group: normal testicular histomorphology. (**C**) Nicotine group: spermatogonial and spermatocytic degeneration with marked cellular disruption in some seminiferous tubules (black arrows), hydropic degeneration of Leydig cells (white arrows), and few mature spermatids in a degenerated tubule. (**D**) Nicotine plus silymarin group: mild degenerative changes in a small number of tubules and hydropic degeneration in occasional Leydig cells. Hematoxylin and eosin staining.

The seminiferous tubules, spermatogenic cell series, and interstitial spaces displayed normal histomorphology in the control and silymarin groups. In the nicotine group, several seminiferous tubules showed spermatogonial and spermatocytic degeneration, fewer mature spermatids, and widespread hydropic degeneration of Leydig cells (observed in 7 out of 7 mice). In the nicotine plus silymarin group, cellular injury in the seminiferous tubules appeared less extensive; mild degenerative changes were confined to a small number of tubules, and hydropic degeneration was observed in occasional Leydig cells (observed in 6 out of 7 mice) (Figure 6).

## 3. Discussion

The main finding was that seven days of nicotine administration disrupted both circulating and testicular redox measures and was accompanied by higher NF-κB and caspase-3 values and histological degeneration. Concurrent silymarin administration attenuated each of these alterations and was associated with less severe histological injury. Because several plasma markers and testicular GSH did not fully return to control values, the observed effect appears partial at the tested dose and duration.

The testis generates reactive oxygen species during steroidogenesis and spermatogenesis. Although low concentrations contribute to cellular signaling, production that exceeds antioxidant capacity promotes lipid peroxidation in germ-cell membranes rich in polyunsaturated fatty acids [11]. This process can impair membrane fluidity, mitochondrial energy production, and cellular integrity. Systematic reviews of human sperm have likewise associated excessive oxidative burden with impaired motility, DNA damage, and dysfunction [12]. The lower GSH and higher MDA observed after nicotine exposure may therefore represent complementary aspects of the same redox disturbance.

The present findings are consistent with earlier experimental nicotine models. Budin et al. reported higher testicular MDA, lower SOD activity, and disrupted testicular architecture after nicotine exposure [2]. Oyeyipo et al. found increased lipid peroxidation and NO with reduced antioxidant defenses in reproductive tissues [3], and Jana et al. described increased testicular lipid peroxidation together with suppressed glutathione, steroidogenic enzymes, and spermatogenesis [4]. Increased testicular oxidative stress and weakened antioxidant defenses have also been reported in nicotine-exposed mice [13].

The higher NO value in the nicotine group suggests that nitrosative stress accompanied oxidative stress. NO contributes to testicular blood flow and cellular communication under physiological conditions, but in the presence of excess superoxide it can promote peroxynitrite formation and further oxidative injury to lipids, proteins, and DNA. Previous reports of increased NO after nicotine exposure [3] support this interpretation. The absence of a significant difference between the nicotine plus silymarin and control groups may indicate attenuation of reactive-species production, but direct reactive-species measurements were not performed.

PON1 is carried mainly on high-density lipoprotein and contributes to the hydrolysis of oxidized lipids [14]. High-density lipoprotein provides an appropriate microenvironment for PON1 secretion, stability, and activity [15]. In this study, PON1 activity was lower after nicotine exposure, although HDL-C and total cholesterol were unchanged. This pattern may reflect oxidative modification, enzyme inactivation, or altered functional composition of high-density lipoprotein rather than a quantitative reduction in the lipoprotein itself. Silymarin increased PON1 activity relative to nicotine alone, although values remained below controls.

Elevated plasma NF-κB and caspase-3 levels in the nicotine group reflect a systemic inflammatory response and enhanced overall apoptotic activity induced by nicotine exposure. However, as these markers were measured in the systemic circulation, they cannot be directly attributed to specific molecular events occurring exclusively within the testicular tissue, particularly given the compartmentalization and immunological privilege imposed by the blood-testis barrier [16,17,18,19]. Therefore, while these systemic markers indicate a generalized pro-inflammatory and pro-apoptotic state, direct conclusions regarding localized testicular inflammation or specific germ cell apoptosis should be interpreted with caution and require confirmation through direct tissue-level molecular analyses.

The higher TSA value after nicotine exposure and its partial attenuation with silymarin may reflect systemic stress involving cell-surface glycoconjugate turnover or acute-phase glycoproteins. TSA is not a specific marker of testicular injury and can be influenced by inflammation, tissue remodeling, and metabolic processes. It should therefore be interpreted as a complementary finding rather than as an independent indicator of reproductive toxicity.

Testicular markers provided target-organ context for the systemic findings. The absence of significant differences in testicular MDA and SOD between the nicotine plus silymarin and control groups suggests attenuation of lipid peroxidation and preservation of enzymatic antioxidant defense. The incomplete recovery of testicular GSH may reflect slower replenishment of the thiol pool or continued consumption during nicotine exposure. Antioxidant-based interventions have improved testicular redox balance and reproductive measures in other nicotine models [13,20,21]. An interesting finding of the present study is the discrepancy in the responses of oxidative stress markers (MDA, SOD, and GSH) between systemic circulation (blood) and the local testicular microenvironment following combined nicotine and silymarin administration. While blood parameters reflect the overall systemic oxidative status, testicular tissue responses are governed by local cellular metabolism and the unique physiological properties of the organ. The observed differences can be primarily attributed to the presence of the blood-testis barrier (BTB), which restricts the free exchange of molecules, including antioxidants and toxins, between the systemic circulation and the seminiferous tubules [16,17]. Furthermore, silymarin’s bioavailability and metabolic activation may differ between hepatic/systemic circulation and the testicular tissue, leading to a more pronounced or delayed protective effect locally compared to the blood. Therefore, the partial dissociation between systemic and tissue-level biochemical changes is not contradictory but rather highlights the compartmentalized nature of oxidative stress and the organ-specific protective mechanisms of silymarin.

Spermatogonial and spermatocytic degeneration, fewer mature spermatids, and hydropic changes in Leydig cells indicate that injury involved both the germinal epithelium and steroid-producing interstitial cells. Similar studies have reported seminiferous-tubule atrophy, germ-cell loss, impaired spermatogenesis, and Leydig-cell changes [2,4,5]. The effects of melatonin on Johnsen score, germ-cell apoptosis, sperm chromatin integrity, and testosterone in nicotine-exposed mice further suggest that oxidative stress, apoptosis, and endocrine disruption may be interrelated [22].

Histological improvement does not establish preservation of fertility. Meta-analyses in men who smoke have reported poorer sperm count, motility, and morphology [23,24], and oxidative stress, DNA damage, and apoptosis have been proposed as contributing mechanisms [25,26]. Smoking has also been associated with impaired sperm-DNA integrity and higher 8-hydroxy-2′-deoxyguanosine [27]. Because sperm count, motility, morphology, DNA fragmentation, mating success, and offspring outcomes were not assessed, the present results should be limited to biochemical and morphological testicular outcomes.

Silymarin may act through several complementary processes. Its flavonolignans may scavenge free radicals, support cellular GSH pools, limit lipid peroxidation, and help preserve mitochondrial membrane integrity [6,28]. Reproductive-toxicity research also suggests potential involvement of nuclear factor erythroid 2-related factor 2-mediated antioxidant responses, lower NF-κB signaling, and reduced apoptosis [7,29]. The present study, however, did not directly assess these pathways in testicular tissue and therefore cannot establish a specific mechanism.

The protective counter-mechanisms of silymarin observed in our short-term interventional window align closely with broader reproductive toxicity frameworks reported in the recent literature. Specifically, Rahimi-Madiseh et al. [10] demonstrated that longer-term, 28-day silymarin administration effectively rescued sperm parameters, cell viability, and in vitro fertilization outcomes from nicotine-induced gonadal degradation in mice. While their model confirmed the preservation of final reproductive performance over a full spermatogenic cycle, our findings capture the immediate phase of this protective architecture, demonstrating that silymarin accelerates the preservation of local tissue thiol pools and structural integrity during the critical early windows of toxic insult. Effects of silymarin on testicular tissue have also been reported in other injury models. In acrylamide-exposed rats, silymarin reduced MDA, improved antioxidant enzymes and sperm parameters, and limited DNA fragmentation and histopathological injury [8]. In diabetic rats, it reduced serum MDA, increased total antioxidant capacity, and improved seminiferous-tubule architecture [9]. Biochemical and histopathological attenuation has also been reported in testicular torsion/detorsion [30]. A meta-analysis of randomized human studies found lower MDA, C-reactive protein, and interleukin-6 with silymarin [31], although those studies did not assess reproductive toxicity and cannot be directly generalized to the testis.

The full recovery of some variables and partial recovery of others may reflect differences in kinetics and biological sources. Circulating GSH and NO may respond rapidly, whereas clearance of lipid-peroxidation products, restoration of PON1, or glycoprotein turnover affecting TSA may require more time. It is also possible that 100 mg/kg silymarin was sufficient for some redox processes but not others. Dose–response and time-series data are needed to distinguish these possibilities.

The absence of group differences in CHOL and HDL-C suggests that the seven-day protocol did not markedly change plasma lipid concentrations. The isolated TG increase in the silymarin group was not accompanied by changes in other lipid measures, was absent in the combined-treatment group, and had a wide standard deviation. With seven animals per group and no reported standardization of fasting status or sampling time, this finding should not be interpreted as evidence of a consistent dyslipidemic effect.

The study is limited by its small group size, short exposure period, and incomplete reporting of treatment preparation, sampling, assay-validation, and blinding procedures. The absence of sperm-function, hormonal, DNA-damage, and fertility outcomes restricts functional interpretation. Plasma NF-κB and caspase-3 do not identify the cellular origin of these signals, and the integrated application of both qualitative histopathological observations and standardized quantitative histomorphometric measurements provides a rigorous evaluation of tissue injury. By utilizing computerized image analysis to determine tubule dimensions and epithelial height, the present study successfully overcomes the limitations of purely subjective scoring, offering a robust assessment of reproductive tissue protection under the tested conditions. The study also lacks dose–response and recovery-period groups. Future work should use fully matched vehicles, preregistered sample-size calculations, blinded quantitative histopathology, multiple doses and time points, and integrated biochemical, molecular, endocrine, sperm, and fertility outcomes.

## 4. Materials and Methods

### 4.1. Experimental Animals and Study Design

Twenty-eight adult male Swiss albino mice, 8–12 weeks of age and weighing 20–30 g at the start of the study, were obtained from the Experimental Animal Application and Research Center of Kafkas University. The mice were housed under standard laboratory conditions at 22 ± 2 °C and 50–60% relative humidity on a 12 h light/12 h dark cycle, with animals allocated across multiple cages to limit social and environmental stress, using pine-shaving bedding. To control for potential cage-associated confounding factors and minimize social density stress, the seven mice within each experimental group were divided and housed in two separate, independent cages (one containing three mice and the other containing four mice) under identical environmental microconditions. Standard pellet feed and drinking water were provided ad libitum. The sample size for this study was determined via an a priori power analysis using G*Power 3.1 software (F tests—ANOVA: Fixed effects, omnibus, one-way). For the analysis, the effect size (Cohen’s f) was set at 0.68, the significance level (α) at 0.05, and the statistical power (1 − β) at 0.80. Based on the four-group study design, the calculation indicated that the minimum required total sample size was 28 mice. Accordingly, a total of 28 mice were manually allocated equally across the four experimental groups by simple randomization. This sample size calculation aligns with the ethical principle of ‘Reduction’ in animal research, ensuring sufficient statistical power while minimizing the number of animals used. No animals exhibited signs of severe systemic illness, and no specific exclusion criteria were triggered during the trial. Consequently, no subjects were excluded, and the actual number of animals included in all final biochemical and histological analyses was exactly seven per group (*n* = 7/group). To ensure scientific objectivity, all blood and tissue samples were assigned arbitrary numbers upon collection, thereby maintaining strict investigator blinding during all subsequent laboratory assays and microscopic assessments. After a one-week acclimatization period, the animals were allocated by simple randomization to four experimental groups of seven animals each (*n* = 7/group) as follows.

Group I—Control (Vehicle): Animals received two concurrent intraperitoneal (i.p.) injections consisting of sterile 0.9% NaCl solution and the silymarin vehicle [propylene glycol and 0.9% NaCl solution (25/75, *v*/*v*)] to serve as a complete cross-matched double-vehicle control.

Group II—Nicotine: Animals received nicotine (1.5 mg/kg/day) dissolved in sterile 0.9% NaCl solution via i.p. injection and concurrently received an equivalent volume of the sterile propylene glycol/saline vehicle.

Group III—Silymarin: Animals received an equivalent volume of sterile 0.9% NaCl solution via i.p. injection and concurrently received silymarin (100 mg/kg/day) dissolved in the sterile propylene glycol/saline vehicle [32].

Group IV—Nicotine plus Silymarin: Animals received concurrent i.p. administrations of nicotine (1.5 mg/kg/day) dissolved in saline and silymarin (100 mg/kg/day) dissolved in the propylene glycol/saline vehicle [32,33].

### 4.2. Chemical Preparations and Administration Regimen

Nicotine (Sigma-Aldrich, St. Louis, MO, USA) was dissolved in sterile 0.9% NaCl solution to achieve a stock working concentration of 0.75 mg/mL. Due to its poor aqueous solubility, silymarin (Sigma-Aldrich, St. Louis, MO, USA) was dissolved in a sterile vehicle consisting of propylene glycol and 0.9% NaCl solution (25/75, *v*/*v*) to achieve a working concentration of 50 mg/mL, ensuring complete solubilization for systemic delivery following validated antioxidant protection protocols. Both solutions were freshly prepared on a daily basis. To rigorously eliminate any confounding variations arising from vehicle components, total fluid load, or injection-induced physical stress, a double-injection cross-matching protocol was implemented across all four experimental groups. Each individual injection was standardized at a fluid volume of 2 mL/kg based on daily body weight measurements, resulting in a strict, uniform total daily fluid administration of exactly 4 mL/kg (2 mL/kg × 2 injections) per animal across all groups. Group IV received concurrent active injections of nicotine (2 mL/kg) and silymarin (2 mL/kg). Groups I, II, and III received their respective active treatments cross-matched with corresponding volumes (2 mL/kg) of sterile saline or the propylene glycol vehicle to maintain absolute baseline uniformity. All administrations were performed at the same time each day, and a strict interval of exactly 24 h was maintained between the final administered dose on day 7 and the subsequent sample collection.

### 4.3. Sample Collection and Preparation

At the conclusion of the experimental period, the animals were deeply anesthetized with a ketamine/xylazine mixture (80/10 mg/kg, i.p.; Ketalar, Pfizer Inc., Istanbul, Turkey and Rompun, Bayer AG, Leverkusen, Germany). Blood samples were collected via intracardiac puncture into heparinized tubes, after which the mice were euthanized by cervical dislocation. The testes were immediately excised. Tissue samples designated for biochemical analysis were homogenized in phosphate-buffered saline (PBS) and stored at −20 °C until analysis, while those allocated for histopathological examination were fixed in Bouin’s solution.

### 4.4. Biochemical Analyses

Whole-blood reduced GSH was measured using the spectrophotometric method of Beutler et al., based on the formation of a colored product between sulfhydryl groups and 5,5′-dithiobis-(2-nitrobenzoic acid) [34]. Plasma MDA was determined by the thiobarbituric acid-reactive substances method of Yoshioka et al. [35]. NO was measured as total nitrate and nitrite using the spectrophotometric method of Miranda et al. [36]. Plasma and testicular SOD activities were determined using the Marklund and Marklund method, which measures inhibition of pyrogallol autoxidation under alkaline conditions [37]. Absorbance was monitored kinetically at 420 nm; the enzyme amount producing 50% inhibition of pyrogallol autoxidation was defined as one SOD unit. Results were expressed as U/mL for plasma and as U/mg protein for testicular tissue. Paraoxonase-1 (PON1) activity was measured using the methods of Eckerson et al. and Gülcü and Gürsu [38,39]. Total sialic acid (TSA) was determined using the Sydow method [40]. Total cholesterol (CHOL), high-density lipoprotein cholesterol (HDL-C), and triglycerides (TG) were measured spectrophotometrically with commercial reagent kits (Spinreact SA, Sant Gregori, Girona, Spain; Ref. 1001090, 1001095, and 1001312, respectively).

Plasma caspase-3 and nuclear factor kappa B (NF-κB) concentrations were measured with a Mouse Caspase-3/CPP32 ELISA Kit (YL Biont, Shanghai, China; Cat. No. YLA0081MO) and a Mouse Nuclear Factor Kappa B ELISA Kit (Cat. No. YLA0079MO), respectively, using sandwich ELISA according to the manufacturer’s instructions. Microplate absorbance was recorded at 450 nm.

Testicular tissue samples were homogenized in cold 0.1 M phosphate-buffered saline (PBS, pH 7.4) using a mechanical homogenizer. The homogenates were then centrifuged at 10,000× *g* for 15 min at 4 °C, and the resulting clear supernatants were harvested for subsequent biochemical assessments. Testicular MDA concentrations and reduced GSH content were determined spectrophotometrically using the exact chemical reaction methodologies described above for plasma and whole blood (the thiobarbituric acid-reactive substances method of Yoshioka et al. [35] and the DTNB sulfhydryl-binding method of Beutler et al. [34], respectively). Testicular SOD enzyme activity was measured via the kinetic pyrogallol autoxidation inhibition method of Marklund and Marklund [37], as detailed previously. To ensure methodological precision and account for tissue mass variations, total protein concentrations in the testicular supernatants were quantified spectrophotometrically using a commercial biuret-based reagent kit (Spinreact SA, Sant Gregori, Girona, Spain). All targeted testicular redox parameters were strictly normalized against the total tissue protein content and expressed as nmol/mg protein for MDA, µmol/mg protein for GSH, and U/mg protein for SOD activity.

### 4.5. Testicular Histopathological Examination

Testicular tissue specimens fixed in Bouin’s solution underwent routine tissue processing to create paraffin blocks of transverse testis sections. Sections with a thickness of 5 µm were cut from these paraffin blocks and stained with hematoxylin and eosin (H&E). The slides were examined and photographed under a high-resolution light microscope (Olympus BX53, Tokyo, Japan) equipped with a digital camera system (Olympus DP72, Tokyo, Japan) for seminiferous-tubule architecture, the appearance of spermatogenic cells, and changes in interstitial Leydig cells. During the evaluation phase, five non-consecutive sections from each testis were meticulously analyzed. These microscopic examinations were conducted in a strictly blinded manner, without any prior knowledge of which experimental group the animals belonged to.

### 4.6. Testicular Histomorphometric Evaluation

For the histomorphometric evaluation of testicular tissues, seminiferous tubule diameter, tubule lumen diameter, germinal epithelial height, and basement membrane thickness were measured using ImageJ software (version 1.54g; National Institutes of Health, Bethesda, MD, USA). For each animal, digital images captured from five distinct tissue sections and five randomly selected microscopic fields per section were evaluated. The arithmetic mean of the measurements was calculated for each animal to determine representative individual values for subsequent inter-group comparative evaluation.

### 4.7. Statistical Analysis

All experimental data obtained from circulating biochemical parameters, peripheral biomarkers, plasma lipid profiles, and localized testicular tissue histomorphometric measurements were compiled and analyzed using SPSS version 26.0 (IBM Corp., Armonk, NY, USA). The datasets were initially evaluated for normality of distribution using the Shapiro–Wilk test, while the homogeneity of variance across the experimental groups was verified via Levene’s test. Once the standard parametric assumptions were fully satisfied, statistical comparisons among the four independent experimental groups were performed using one-way analysis of variance (one-way ANOVA). Post hoc pairwise comparisons between specific groups were subsequently executed using Tukey’s honestly significant difference (HSD) test. Quantitative values are expressed as mean ± standard deviation (SD) for exactly seven mice per group (*n* = 7). Statistical significance for all evaluated metabolic and structural pathways was stringently defined at *p* < 0.05. Within the text, tables, and figures, group means displaying different superscript letters indicate statistically significant differences.

## 5. Conclusions

In conclusion, short-term silymarin administration significantly attenuated nicotine-induced oxidative stress and biochemical alterations in mice. However, it is important to note that while the combined treatment significantly mitigated nicotine-induced damage, certain parameters—specifically plasma MDA and TSA concentrations, plasma SOD and PON1 enzyme activities, and testicular GSH content—remained significantly different from the control group, indicating that full biochemical restoration was not achieved within the 7-day exposure period. Furthermore, the conclusion regarding less severe histological injury in the combined-treatment group is fully substantiated by the added quantitative histomorphometric analysis. Our detailed morphometric results precisely quantify the extent of this structural protection, confirming that silymarin successfully limits the reduction in seminiferous tubule diameter and germinal epithelium height induced by nicotine exposure.

## Data Availability

The data presented in this study are available from the corresponding author upon reasonable request. The data are not publicly available due to ethical restrictions concerning raw animal experimental data.

## Abbreviations

CHOL	total cholesterol
GSH	glutathione
H&E	hematoxylin and eosin
HDL-C	high-density lipoprotein cholesterol
MDA	malondialdehyde
NF-κB	nuclear factor kappa B
NO	nitric oxide
PON1	paraoxonase 1
SOD	superoxide dismutase
TG	triglyceride
TSA	total sialic acid
